# Coronary Artery Disease, Family History, and Screening Perspectives: An Up-to-Date Review

**DOI:** 10.3390/jcm13195833

**Published:** 2024-09-30

**Authors:** Francesca Di Lenarda, Angela Balestrucci, Riccardo Terzi, Pedro Lopes, Giuseppe Ciliberti, Davide Marchetti, Matteo Schillaci, Marco Doldi, Eleonora Melotti, Angelo Ratti, Andrea Provera, Pasquale Paolisso, Daniele Andreini, Edoardo Conte

**Affiliations:** 1 Department of Medical and Surgical Sciences, Faculty of Medicine and Surgery, School of Cardiovascular Diseases, University of Milan, 20126 Milan, Italyangelabalestrucci@yahoo.it (A.B.); riccardo.terzi@unimi.it (R.T.); 2 Department of Cardiology, Hospital de Santa Cruz, Centro Hospitalar Lisboa Ocidental, Carnaxide, 2799-134 Lisbon, Portugal; pedro_fagalopes@hotmail.com; 3 Department of Medical and Surgical Sciences, Faculty of Medicine and Surgery, School of Cardiovascular Diseases, Marche University Hospital, 60121 Ancona, Italy; giuseppe.ciliberti@ospedaliriuniti.marche.it; 4 Department of Medical and Surgical Sciences, Faculty of Medicine and Surgery, School of Cardiovascular Diseases, Ospedale Galeazzi-Sant’Ambrogio, 20157 Milan, Italy; davide.marchetti@grupposandonato.it (D.M.); m.schillaci91@gmail.com (M.S.); marco.doldi@grupposandonato.it (M.D.); eleonora.melotti@grupposandonato.it (E.M.); angelo.ratti@grupposandonato.it (A.R.); andrea.provera@unimi.it (A.P.); pasquale.paolisso@grupposandonato.it (P.P.); daniele.andreini@unimi.it (D.A.)

**Keywords:** coronary artery disease, family history for CAD, cardiac CT, high-risk plaques

## Abstract

Family history for CAD (coronary artery disease) is an established cardiovascular (CV) risk factor and it is progressively acquiring importance in patients’ CV risk stratification. Numerous studies have demonstrated that individuals with a first-degree relative affected by CAD have a significantly higher risk of developing the condition themselves; in particular, when CAD occurs at an early age in relatives. Indeed, recently published CCS (chronic coronary syndrome) ESC (European Society of Cardiology) guidelines include family history (FH) as a risk factor to consider when calculating pre-test risk for CAD. ESC guidelines on preventive cardiology (2021) only suggested CV risk assessment in the presence of a positive FH for CV disease, not considering it in the actual risk scores. Evidence suggests that positive anamnesis for relatives affected by CAD correlates with ACS (acute coronary syndrome) and CAD, with slight differences in relative risk as far as the degree of kinship is concerned. Genetic factors contribute to this correlation by influencing key processes that affect heart health, such as cholesterol metabolism, blood pressure regulation, and inflammatory responses. New technologies in the genetics field are increasing the availability of genome sequencing, and new polymorphism panels are being tested as predictive for CAD, objectifying familiarity. Advances in imaging techniques allow the assessment of coronary atherosclerosis and its composition, and these are acquiring strength in evidence and recommendations in ESC guidelines as a way to define coronary disease in low and low-to-intermediate risk patients and to guide medical therapy and interventional procedures. Use of these emerging tools to guide screening is likely to be extended, beyond high CV risk patients, to individuals with FH for early CAD and/or specific genetic profiles, as recent evidence in the literature is suggesting.

## 1. Background

Coronary artery disease (CAD) is the most common cause of mortality and morbidity worldwide, accounting for 16% of all deaths, resulting from the complex interplay of a person’s individual environmental, lifestyle, and genetic factors [1]. According to WHO (2020), CAD is responsible for around 9 million deaths annually; according to the Global Burden of Disease Study (2021), it affects 126 million people worldwide (about 17.2% of the global population).

To date, available cardiovascular (CV) risk scores (SCORE, ESC 2016-; PCE, AHA 2019-; Framingham Heart Study Risk Score and QRISK3, NICE 2023) do not include family history (FH) for CAD as a variable kept in consideration to evaluate CV risk, and it is instead considered a “Risk Modifier” along with psychological and social factors, BMI, and ABI. The only risk score partially including the above-mentioned is QRISK3, which considers FH for acute coronary syndrome [2,3,4].

**Figure 1:**
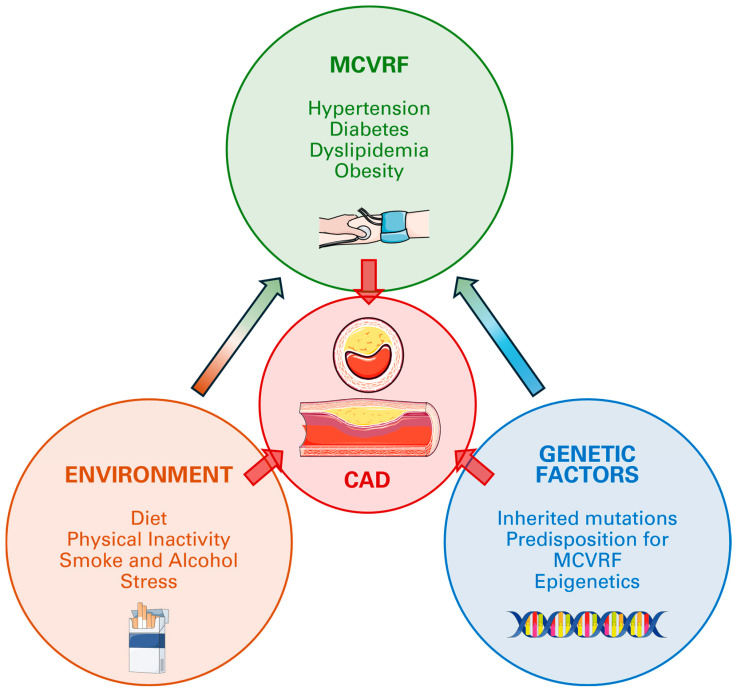
Factors involved in CAD development.

Mechanisms underlying the higher prevalence of CAD in patients with positive anamnesis for relatives affected by CAD include inherited pathologies (familial hypercholesterolemia), genetic predisposition (affecting the development of atherosclerosis via inflammation, blood pressure regulation, and cholesterol metabolism), inherited risk factors (such as diabetes, hypertension, and dyslipidemia), shared lifestyle and environment (diet, smoking, and physical inactivity), and epigenetics (Figure 1).

Although it is generally accepted that the risk of developing CAD at a young age is significantly higher among first-degree relatives of affected patients, especially among siblings [5,6], and that there is a correlation between early CAD and coronary artery calcification [6] (considered an indirect indicator of atherosclerosis), the role of family history as an independent risk factor for CAD has not been fully clarified (unlike other risk factors such as hypertension, diabetes, and dyslipidemia, whose roles in the etiopathogenesis of coronary disease have been extensively studied). For this reason, FH of early CAD is not considered in most major cardiovascular risk assessment models. Nevertheless, emerging evidence is confirming the pivotal role FH covers in determining CAD and acute coronary syndromes (ACSs) [7,8].

Recently published CCS ESC guidelines include FH for CAD as criteria to keep in consideration when calculating risk factor-weighted clinical likelihood (RF-CL) during pre-test assessment, testifying to the importance of this anamnestic record [9].

Coronary Computed Tomography Angiography (CCTA) is emerging as a method of screening for the presence of CAD in subjects with low–intermediate pre-test probability, currently limited to coronary artery calcium (CAC) evaluation, and has recently showed high accuracy in characterizing plaque composition [10,11,12].

The aim of this review is to outline up-to-date evidence of FH for CAD in defining a patient’s CV risk, potential future screening options, and early therapeutic strategies. Reported below are the main published studies regarding FH and coronary artery disease (Table 1).

## 2. Framingham Heart Study

One of the most important and long-running epidemiological studies on CAD is the Framingham Heart Study (FHS). This study has demonstrated that a positive FH for CAD is associated with an increased risk of developing CAD. Thanks to data collected over decades, the Framingham study has identified numerous risk factors for CVDs and highlighted the importance of primary prevention in reducing the incidence of these diseases. This study has a pioneering role in pointing out FH for CAD as a CAD determinant [22,23]. Subsequent studies on nurses and doctors confirmed that having first-degree relatives affected by CAD increases the risk of developing the disease, with a higher risk if CAD manifests early in the relatives (defining as premature CAD). Moreover, the Framingham Offspring Study demonstrated that a paternal history of premature CVD, including coronary death, myocardial infarction (MI), angina, ischemic stroke, and intermittent claudication is as important as maternal history in determining CVD risk in both men and women [19,22].

One of the most significant contributions of the study was the creation of the Framingham risk score, a predictive model used to estimate the risk of developing CVDs based on various risk factors. This index is widely used in clinical settings to assess individual risk and guide therapeutic decisions and does not include FH. The identification of a correlation between FH and CAD in the context of the Framingham Heart Study has also facilitated genetic research. Family sampling has enabled heritability analysis, linkage, and genome-wide association studies (GWAS) for relevant clinical traits. These analyses have contributed to advancements in the molecular epidemiology of cardiovascular diseases, including CAD.

Among the studies conducted to identify the role of family history in cardiovascular risk stratification, efforts have been made to determine if family history could further improve the stratification of individuals into clinically relevant risk categories based on the Framingham Risk Score (FRS). A study conducted on the cohort of patients from the prospective EPIC-Norfolk study examined whether adding the family history of premature CHD to the FRS could improve cardiovascular risk stratification. However, the analysis revealed that this addition did not lead to an overall improvement in the prediction of CHD risk in the entire cohort. Despite this, in the subgroup of individuals classified as intermediate risk according to the FRS, the inclusion of a family history of premature CHD resulted in a modest improvement in risk classification [21].

## 3. Evidence from Genetics Analysis

Family history for MI has proven to be one of the major cardiovascular risk factors [17]. The basis of this correlation is currently under investigation: familial environment and consequent exposure to the same MCVRFs, epigenetics, and genetics have all been questioned as potential influences. Hypertension, diabetes type II and dyslipidemia are clearly characterized by a certain grade of hereditability and dietary habits and tabagism are very often shared in the familiar environment. Despite this, an independent correlation of several genes has been proved.

As far as monogenic mutations are concerned, the greatest interest is towards those responsible for familial hypercholesterolemia (mutations in LDLR/APOB/PCSK9). The prevalence of this condition has been proved to be 18-fold higher (1:17) among patients with atherosclerotic cardiovascular disease than among the general population [24]. Nevertheless, monogenic mutations are rare in the general population and explain only a small fraction of disease cases.

Specific risk scores (polygenic risk scores, PRSs) have been developed to directly identify the shared polygenic component of FH and its implication in the onset of CVDs (and other diseases). PRSs are designed to predict individual genetic predisposition to disease risk. These combine contributions from several genes with moderate or weak effects, unlike Mendelian traits caused by variations in a single gene. Thanks to large-scale genomic projects and low-cost genotyping platforms, genome-wide association studies (GWASs) have identified thousands of loci associated with complex traits and diseases [25]. Since FH includes both genetic and environmental components, we can expect that PRSs may better define common genetic components, independently from shared environment or monogenic components. Consequently, for predictive purposes, creating PRSs based on anamnestic FH for CAD would likely be more effective than using either factor alone. PRSs have become a powerful tool for predicting genetic predisposition to diseases. Integrating them into existing risk models has improved disease risk prediction accuracy, influencing clinical management. Although questions remain about the ultimate utility of PRSs, their practical implementation as a minimally invasive genetic test shows potential in guiding screening programs, therapies, and lifestyle recommendations [25]. PRS accuracy is expected to improve when involving larger cohorts.

One of the most significant developments in CHD risk prediction has been the introduction of metaGRS, a combined genetic risk score based on data from the largest previous genome-wide associations for CHD. A study on nearly 500,000 people showed that metaGRS has a greater ability to discriminate risk compared to previous genetic scores, with a fourfold risk ratio between individuals in the highest and lowest quintiles. This genetic score was largely independent of traditional CHD risk factors, suggesting that genetic information can complement rather than replace conventional risk factors. Moreover, metaGRS was particularly effective in identifying individuals at high risk of premature CHD, which would offer the opportunity for more intensive preventive interventions [26].

In addition to metaGRS, other studies have confirmed the importance of GRS in CHD risk prediction. An analysis of 23,595 participants highlighted that CHD risk assessed through the GRS is independent of self-reported FH, with higher risk estimates in younger individuals compared to older ones. This suggests that the GRS can be particularly useful for risk assessment in young adults and can be beneficial in clinical practice to intensify early medical therapy [27].

The importance of genetic testing has been further highlighted by a study involving patients hospitalized for early myocardial infarction (MI). Both familial hypercholesterolemia mutations and polygenic scores were associated with a significant increase in early MI risk. Specifically, patients with high polygenic scores were identified at a tenfold higher proportion compared to those with monogenic mutations [28]. These results clearly indicate that genetic testing can play a crucial role in identifying high genetic risk individuals. Furthermore, statin therapy has been particularly effective in patients with high genetic risk for CAD. Despite similar reductions in LDL cholesterol levels, statin therapy led to a significant reduction in relative risk in patients with high genetic risk. Moreover, subclinical atherosclerosis was more pronounced in young and middle-aged patients, suggesting that early identification of genetic risk could allow targeted therapeutic interventions to prevent disease progression [29].

Finally, the development of a genetic risk score (GRS) based on SNP for CAD has further expanded our knowledge. This tool has demonstrated an improved 10-year CHD risk prediction and has been associated with CAD independently of established clinical risk scores [30,31].

Therefore, genetic testing can enhance the early detection of coronary artery disease (CAD) and refine cardiovascular risk assessment by identifying individuals with a genetic predisposition. However, these tests are often expensive and may not be widely accessible, especially in low-resource settings. This limits their utility in routine clinical practice, making it challenging to implement them universally for early CAD screening and personalized risk management in most clinical realities.

## 4. Degree of Kinship in Family History and Evidence of Coronary Artery Disease

Understanding family history and the degree of kinship is a crucial step in evaluating an individual’s risk of CAD. This assessment provides valuable guidance for making preventive and therapeutic decisions, allowing individuals to adopt a healthy lifestyle and undergo regular check-ups to prevent cardiovascular events.

The influence of kinship degree on the likelihood of CV events also depends on other risk factors, such as age, sex, lifestyle, and the presence of concomitant medical conditions like hypertension, diabetes, and dyslipidemia. For example, if an individual has a family history of CAD and other CV risk factors, their overall risk will be higher compared to someone without a family history of CAD but with the same risk factors. Nevertheless, as pointed out in the INTERHEART study, adjusting the analysis for the nine risk factors (smoking, lipids, hypertension, diabetes, obesity, diet, physical activity, alcohol consumption, and psychosocial factors), the OR of MI associated with a PH of MI in either parent was 1.81, suggesting that these do not completely justify the risk of MI [17].

Evidence from various international studies has shown that family history, particularly among siblings, influences the risk of AMI and subclinical atherosclerosis [5,6]. For example, a study conducted in Denmark focused on the risk of myocardial infarction in first-degree relatives of patients with AMI. The results showed a significantly higher risk for siblings of a patient with MI and for children whose mother had an MI. When parents had an MI before the age of 50, the risk of MI for the children was particularly high [5,6]. This study demonstrated that only part of the risk can be explained by common risk factors.

An analysis conducted in the United States suggested that descendants of a patient with MI have a lower prevalence of the disease compared to the siblings of a patient with MI. It is believed that shared genetic and environmental factors may explain the elevated risk for siblings of a patient with MI, especially if the mother is affected [5,6].

It has also been highlighted that the onset of CVDs in parents is an independent predictor of CV events in their middle-aged offspring. Adjusting for other risk factors, the presence of early CVD in at least one parent is associated with a significant increase in CV risk in men and a 70% (non-statistically significant) increase in risk in women [19]. This association was observed mainly in subjects with intermediate levels of cardiovascular risk. However, the association between parental CVD and offspring CVD was attenuated by adjusting for age and other traditional risk factors.

Other studies have also highlighted how the relationship between FH and the risk of MI varies based on the number of affected relatives, the sex of the relative involved, and the age at which events occur [13,14]. These findings provide important guidance for clinicians in evaluating patients’ risk and therapeutic decisions, suggesting that including FH can improve risk prediction, especially for patients with intermediate CV risk. Knowledge of the presence of CVD in parents may not significantly alter the risk level for those who already have very high or very low risk. However, for patients with intermediate risk, this additional information could influence the post-test probability enough to justify a change in treatment and lifestyle [19].

An inception cohort of the Framingham Heart Study demonstrated that the presence of CVD in siblings is correlated with a significant increase in the risk of CVD in middle-aged adults [20]. This risk remains elevated even after adjusting for age, sex, and other traditional risk factors, suggesting that part, but not all, of the risk can be explained by these factors. Compared to a history of parental CVD, the history of CVD in siblings seems to confer a higher risk of CVD events. This occurs because siblings share a closer combination of genetic and environmental factors than parents. These findings suggest that shared genetic and environmental factors among siblings can influence susceptibility to CVD, beyond traditional risk factors. Therefore, acknowledging the history of CVD in siblings can provide valuable additional information for assessing individual CHD risk and making clinical decisions regarding prevention and treatment.

## 5. Acute Coronary Syndromes and Family History for Coronary Artery Disease

In the context of coronary heart disease (CHD), it is essential to distinguish between forms that present acutely and those with chronic manifestation. While both share a genetic component that can influence individual predisposition to CAD, this component plays different roles in the two clinical forms.

Numerous studies have been conducted to evaluate the correlations between FH and acute myocardial infarction (AMI), the main clinical manifestation of acute coronary syndrome (ACS). Among these studies, the INTERHEART study played a significant role in analyzing the association between FH for AMI and the risk of acute myocardial infarction (AMI). This association has been confirmed across various geographic regions, age groups, genders, and socioeconomic subgroups [17]. The relationship between FH for AMI and the risk of AMI is attributed to a combination of shared risk factors and genetics. More detailed information on FH (e.g., considering the number of relatives with coronary disease, the degree of kinship, the line of descent, and age at diagnosis) further improves risk prediction. Other factors, such as early life exposures and household environmental factors contribute to this association, as mentioned above [17].

An investigation analyzed atherosclerotic cardiovascular diseases (ASCVDs) and revealed that a positive FH for the early onset of ASCVD is associated with an increased risk of recurrent ASCVD following AMI, regardless of numerous known cardiovascular risk factors. This highlights the importance of carefully evaluating FH in managing patients who have survived an initial coronary event, as it can be a crucial indicator of the risk of recurrent CV events [7].

Similarly, another study focused on the association between FH for CAD and the risk of ACS in patients with chest pain. The results indicated that a positive anamnesis for the above-mentioned is correlated with an increased risk of ACS, especially when the disease manifests early in relatives. This suggests that the age of onset of the disease in relatives may influence individual risk of ACS [8].

Additionally, the analysis of high-sensitivity cardiac troponin (hs-cTnT) levels showed that, despite normal or non-elevated initial levels, patients with a positive FH for CAD had a significantly increased risk of ACS. This underscores the importance of considering family history as an additional risk factor in evaluating patients with chest pain in the emergency department, especially when cardiac marker results may not be conclusive [8].

## 6. Computer Tomography Screening Role in Patients with Family History for Coronary Artery Disease

A study conducted by Kral et al. (2014) [10] analyzed a group of asymptomatic individuals with a family history of early CAD, using both coronary angiography and coronary artery calcium (CAC) scanning. Among the participants, 45% had coronary plaques, of which 5% were non-calcified. The study highlights that the presence of plaques was more frequent with advancing age, in males, and in the presence of traditional risk factors. Specifically, among those with a strong family history of CAD in siblings, 84% showed coronary plaques, compared to 63% with a less significant history. These results indicate a potential benefit of CAD screening in patients with a family history of early CHD but emphasizes the need for further studies to define the optimal timing and method of conducting this screening [1,10].

Among the proposed screening methods in the study is coronary calcium scanning (CAC), which allows non-invasive detection of coronary atherosclerosis and considers various risk factors [1,32]. In individuals without previous CAD, the presence of high CAC indicates high risk, while the absence of CAC suggests low risk, with an annual clinical event rate of around 0.1%. Studies by Nasir et al. [6,33]. have shown that a family history of early CHD increases the prevalence and severity of coronary calcifications, regardless of other risk factors. Specifically, the presence of coronary plaque (CAC > 0) was 64% in subjects with a family history of early CHD in both parents and siblings, compared to 51% if only one parent was involved, and 40% in the absence of a family history of coronary disease.

Non-contrast computed tomography (CT) with ECG-gating is commonly used to assess CAD risk in high-risk populations but has limitations in detecting non-calcified plaque (NCP), which is a precursor to CAD events. Since families with early CAD have an elevated risk of cardiac events, and the early stages of atherosclerosis are characterized by non-calcified (NCP) or mixed plaques, which are more prone to rupture and clot formation, the presence of NCP has crucial implications for the primary prevention of cardiac events [10]. High-risk patients, such as those with familial dyslipidemia, might be started directly on pharmacological treatment without further tests, as suggested by the guidelines. In light of this, family history should be carefully considered to assess individual risk. For example, a family history of premature cardiovascular disease (i.e., in first-degree male relatives before age 55 or first-degree female relatives before age 65) can refine risk analysis for patients who do not fall into the main groups benefiting from statins. When statin therapy is uncertain, the CAC score could be useful for patient-centered decision making, especially for those reluctant to pharmacotherapy. CAC screening can better identify individuals at high risk of cardiovascular events, distinguishing them from those at low risk who might not benefit from treatment [1].

Another useful diagnostic method for detecting calcified and non-calcified plaques is coronary computed tomography angiography (CCTA). However, it is more expensive than CAC scanning and requires intravenous contrast. Although it is not recommended for screening asymptomatic individuals, since Kral et al.’s study showed that calcified plaque constitutes only part of the total plaque burden, using CTA in a selected group of individuals, such as young subjects with non-calcified plaques, might be advantageous for improving clinical management [10]. Understanding the atherosclerotic process could suggest the potential benefit of treating the early stages of the disease to prevent the progression of CAD and the development of cardiovascular events. However, there is a lack of data demonstrating the real efficacy of such interventions. The early stages of atherosclerosis, preceding the development of coronary calcifications, are difficult to identify, making the CAC test less useful for individuals under 40 years old. Moreover, identifying these early forms could involve low-risk individuals, and the cost–benefit ratio of screening might make this diagnostic approach unfeasible, with economic and clinical–biological costs potentially outweighing the benefits of early diagnosis [1]. Therefore, screening tests should be performed only if the results would influence patient management, leading to increased therapy or lifestyle changes.

As is known, high-risk plaque burden is the strongest predictor of fatal or non-fatal MI, more than stenosis severity. In the multicenter SCOT-HEART trial, patients with a low-attenuation plaque burden >4% were five times more likely to suffer from AMI [11]. Moreover, the CAPIRE study confirmed the prognostic value of atherosclerosis assessment by coronary CTA, demonstrating high non-calcified plaque burden as the most ACS-predictive parameter in patients with extensive CAD [12]. Phenotypic characterization of the plaque aims to define whether a plaque is at high risk. Positive remodeling, spotty calcification, low-attenuation plaque, and the Napkin-ring sign are all indicators of a high-risk plaque. Furthermore, lesion volume progression is an independent predictor of MACE in subjects with unrevascularized non-culprit intermediate stenosis (50–69%) [34].

**Figure 2:**
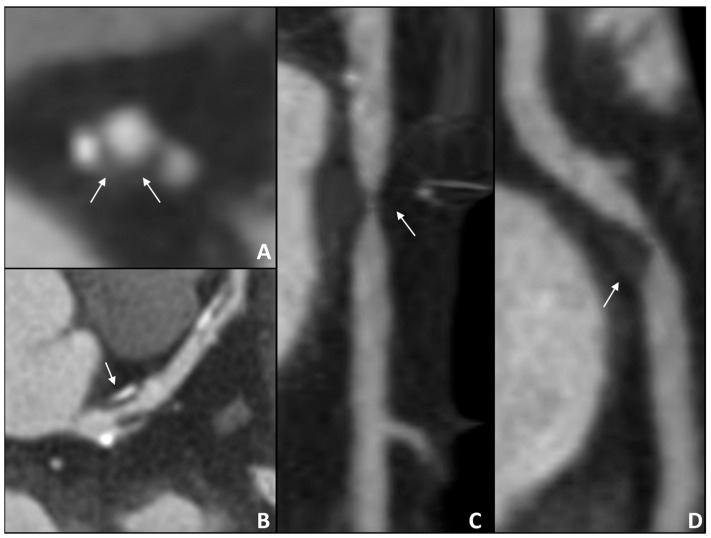
Examples of high-risk plaque features. (**A**,**B**) White arrows show an example of Napkin-ring sign; (**C**,**D**) white arrows show an example of low-attenuated plaque (LAP) with positive remodeling.

Therefore, CCTA can be useful for examining the actual extent of coronary plaque (Figure 2), above all NCP, in asymptomatic members of families with early CAD. This approach can help early evaluation of the risk of CV events and guide clinical management more effectively [10].

## 7. Family History for Coronary Artery Disease and Vascular Disease

Numerous studies have investigated the role of FH in CVDs, demonstrating its variable impact on different vascular districts. In particular, it has emerged that coronary and carotid atherosclerosis, while sharing common risk factors, are distinct diseases influenced differently by genetics. FH for CAD is more associated with significant coronary stenosis than with increased carotid intima-media thickness (IMT). Furthermore, there is a discrepancy between the association of FH for CHD with coronary and carotid atherosclerosis. This suggests that FH for CHD might more likely be linked with coronary atherosclerosis and ACS rather than carotid atherosclerosis and ischemic stroke [35]. The results show that an FH of CAD adds significant predictive value for coronary stenosis, but not for carotid atherosclerosis, suggesting a greater efficacy of coronary pre-screening tests compared to carotid ultrasound in individuals with a family history of first-degree coronary disease [35].

## 8. Limitations of Considering Family History for Coronary Artery Disease as Cardiovascular Risk Factor

The relationship between an FH of premature CAD and major modifiable cardiovascular risk factors (MCVRFs) is complex and interconnected. MCVRFs include hypertension, dyslipidemia, diabetes, obesity, smoking, and physical inactivity. This relationship can be influenced by various biases, further complicating interpretation and clinical application. While FH of premature CAD is an important independent risk factor for CAC and CAD, its assessment can be influenced by biases associated with MCVRFs. Improving the accuracy of FH data and better understanding the interaction between FH and MCVRFs can help provide a more accurate and personalized risk assessment for cardiovascular disease prevention.

The misclassification bias can significantly influence study results and their clinical interpretation; as follows, it consists of:

- Sensitivity and specificity of FH: The sensitivity of a reported FH for premature CHD ranges from 68% to 86%, while specificity is higher (86% to 98%); this implies that some individuals with a positive FH of CHD are erroneously classified as negative (false negatives), leading to an underestimation of the risk associated with FH.
- Self-reporting and memory: Collecting information on FH often relies on self-reporting, which can be influenced by memory errors or a lack of knowledge about relatives’ medical conditions. This leads to recall bias, reducing the accuracy of FH data [33].
- Inaccurate measurement: Misclassification can also occur in MCVRFs themselves: if these are not correctly identified, it can be difficult to determine the independent effect of FH on CAD risk.

The interaction bias between CAD FH and MCVRFs represents another significant challenge in assessing CV risk. Recognizing and managing these interactions is crucial to improving the accuracy of risk assessments and optimizing prevention and treatment strategies.

- Independence of FH from MCVRFs: when premature CAD FH coexists with several MCVRFs, it may be difficult to discern the exact contribution of FH compared to MCVRFs in determining CAD risk.
- Cumulative risk: Individuals with premature CAD FH often share environmental and behavioral risk factors with their relatives, such as dietary habits, physical activity levels, and smoking tendencies. These factors can cumulatively increase the risk of CAD, making it complex to isolate the impact of FH from MCVRFs [33].

Confounding bias is one of the main challenges in epidemiological research because it can distort the true relationship between FH of CHD and MCVRFs. It is essential to recognize and control for confounders through appropriate statistical methods and study design to achieve more accurate and reliable results.

- Confounding from unmeasured factors: Unmeasured genetic or environmental factors can influence both FH and MCVRFs; genetic variants that predispose to dyslipidemia or hypertension increase CAD risk independently of FH.
- Heritability of MCVRFs: Some MCVRFs, such as hypertension and diabetes, have a significant genetic component. Therefore, an FH of premature CAD might partly reflect the heritability of these MCVRFs rather than an independent genetic risk for CAD [33].

Lastly, selection bias can significantly affect the generalizability of epidemiological and clinical study results. It is crucial that studies include a diverse population and that risk assessment models are adapted to reflect ethnic and genetic differences. For example, the Framingham Risk Score (FRS) was developed primarily in a white population and might not be fully applicable to minority ethnic populations. Although the FRS has been noted to work well in the African American population, there is little evidence regarding its utility in other minorities. Therefore, further adjustments to the FRS are needed to better characterize the absolute baseline risk in other population subgroups [33].

## 9. Clinical Diagnostic and Treatment Implications

The 2019 ESC guidelines for the diagnosis and management of chronic coronary syndromes underline the evolving role of CCTA, markedly in low-to-intermediate CV risk profiles, as an emerging screening method for CAD. Given the evidence above-mentioned about the correlation between FH for CAD and coronary atherosclerosis and, more importantly, between FH and ACS, CCTA could cover an even more significant role in screening out the presence of HRPs and, in general, of CAD in patients with a positive FH. Furthermore, including the FH for CAD in risk scores would modify up-to-date risk profiles and this would likely change indications to perform CCTA.

As far as therapy is concerned, the PARADIGM study outlined statins’ association with the slower progression of overall coronary atherosclerosis volume, increased plaque calcification, and a reduction in high-risk plaque features, inducing phenotypic plaque transformation [36]. Therefore, given that individuals with a positive FH for CAD correlate with higher rates of ACS as well as higher rates of dyslipidemia, an early start of lipid-lowering medical therapy could be considered in the near future. Moreover, in patients treated with statins, baseline plaque characterization by plaque burden and HRP is associated with atherosclerotic statin nonresponse: those with the highest plaque burden and HRPs are at highest risk for plaque progression, despite statin therapy. These patients may need additional therapies for further risk reduction [37].

Further studies are required to prove whether analyzing plaque burden and plaque composition in patients with an FH for CAD may be useful in guiding early intervention and optimizing prevention.

## 10. Conclusions

Positive anamnesis for relatives affected by CAD has shown a close correlation to developing ACSs and CAD in general.

An initial integration of FH as a risk factor in the pre-test calculation for CAD has been brought forward in current ESC guidelines for CCS. Further studies may identify FH as the main cardiovascular risk factor sufficient on its own to justify early screening and adopt more aggressive preventive strategies to improve overall health, such as having patients undergo a coronary CT scan when in the presence of a positive FH for CAD.

Integrating genetic data into CV risk assessment models could significantly enhance our ability to identify and manage high-risk patients, objectifying familiarity. Future studies should continue to explore the interaction between genetic, environmental, and traditional factors to develop more effective prevention strategies, such as strict dyslipidemia treatment and SAPT (single antiplatelet therapy) introduction.

## Figures and Tables

**Table 1:** Main published studies regarding FH and coronary artery disease.

Authors, Year	Population (n)	Median Age	Males %	Study Design	Primary Endpoint	Results
GISSI-EFRIM Investigators, 1992 [13]	2022 (916 cases, 1106 controls)	56	88%	Case–control study	Myocardial infarction	FH of AMI is an independent risk factor for MI; no. of relatives and age at AMI is related to OR.
Argentine FRICAS Investigators, 1997 [14]	1060 (cases, 1071 controls)	40	/	Case–control study	Myocardial infarction	OR for any family history of AMI was 2.83 in women; 2.01 in men. Similar OR if the mother (1.98), the father (2.13), or a sibling (2.48) had had an AMI.
Wang Yunyun et al., 2014 [15]	230 (86 young cases, 65 old cases, 79 controls)	40 (young), 69 (old)	69%	Case–control study	STEMI	Young STEMI pts compared to old had more frequent FH of early CAD (54.65 vs. 18.46%; *p* < 0.01); logistic regression analysis showed association of FH of early CAD (OR 3.194) with STEMI in young pts.
Andre R.M. Paixao et al., 2014 [16]	2390	45	95%	Population cohort study	Composite of CHD-related death, AMI, PCI	In multivariate models adjusted for traditional risk factors, FH was independently associated with CHD (HR 2.6). FH and CAC were additive.
Mia Nielsen et al., 2013 [5]	259.613	40–50	/	Retrospective register-based cohort study	Myocardial infarction	FH for MI raises risk for MI in patients, especially if with maternal or sibling FH.
Dallas Heart Study, 2007 [16]	1824 (young)/919 (older)	40 (young), 55 (older)	66% (young), 34% (older)	Population-based probability sample		FHMI is a more important predictor of CAD in young compared with older adults; among young, in those with multiple CVRFs.
Khurram Nasir et al., 2004 [6]	8549	48	69%	Cross-sectional study	Subclinical Atherosclerosis documented at CT scan	FH (and more specifically sibling FH) for premature CHD associates with CAC.
INTERHEART study, 2011 [17]	12,149 cases/14,467 controls	57	74%	Case–control study	Myocardial infarction	FH positively adjusted for MCVRFs correlates with premature MI (OR 1.84)
Hamza Sunman et al., 2013 [18]	349	58	58%	Retrospective cohort study	Non-calcified atherosclerosis at MDCT coronary angiography	FH of premature CAD is associated with severity, extent, and non-calcified CAP at CT
Agnes Wahrenberg et al., 2021 [8]	25,615	62	72%	Register-based cohort study	Recurrent ASCVD after first MI	FH of early ASCVD is associated with recurrent ASCVD after MI, independently of traditional MCVRFs
Donald M. Lloyd-Jones et al., 2004 [19]	2302	44	49%	Prospective epidemiologic cohort study	CVD events	FH for at least one parent with premature CVD has greater risk for CV events (OR 2 for men, 1.7 for women)
Joanne M. Murabito et al., 2005 [20]	5479	51	46%	Prospective epidemiologic cohort study	CVD events	Sibling CVD confers increased risk of CVD events above and beyond established risk factors.
EPIC-Norfolk study, 2010 [21]	22,841	58	45%	Prospective cohort study	CHD (unstable angina, stable angina, MI)	FH of CHD is an independent risk factor of future CHD.
Brian G. Kral et al., 2014 [10]	805	51	44%	Retrospective cohort study	Subclinical CAD detected at coronary CT	Apparently healthy men and women from families with early-onset CAD have a high prevalence of subclinical CAD, composed primarily of non-calcified plaque.
